# ABA and MeJA Induced Catechin and Epicatechin Biosynthesis and Accumulation in *Camellia oleifera* Fruit Shells

**DOI:** 10.3390/plants13162211

**Published:** 2024-08-09

**Authors:** Shucan Liu, Zhaotong He, Huangping Yin, Yue Zhang, Zexuan He, Xiaoxiao Zou, Yan Yin, Fenglin Chen, Xinhong Guo

**Affiliations:** 1 College of Biology, Hunan University, Changsha 410082, China; liushucan231@163.com (S.L.); yinhuangping@hnu.edu.cn (H.Y.); oneplusone_moon@163.com (Y.Z.); 18874038698@163.com (Z.H.);; 2 Chongqing Research Institute, Hunan University, Chongqing 401120, China; 3 State Key Laboratory of Plant Diversity and Specialty Crops, South China Botanical Garden, Chinese Academy of Sciences, Guangzhou 510650, China

**Keywords:** *Camellia oleifera*, fruit shell, resource utilization, catechins

## Abstract

*Camellia oleifera* Abel, one of the most valuable woody oil plants, has been widely cultivated for extracting edible oil. The shell of *C. oleifera* is a by-product generated in the processing of edible oil extraction. However, there is still limited research on the maturity and high-value resource utilization of shell by-products. We found that the *C. oleifera* ‘Huashuo’ (HS) fruit shells contained a high content of catechins. Abscisic acid (ABA) and methyl jasmonate (MeJA) enhanced the accumulation of catechins in *C. oleifera* fruit shells, providing a basis for production and application of the catechins in fruit shells of *C. oleifera*. We further found that 500 μM ABA and 900 μM MeJA significantly promoted the accumulation of catechin (C) and epicatechin (EC) in fruit shells. Following treatment with 900 μM MeJA, the expressions of *CoPAL1*, *CoC4H1*, *CoC4H2*, *CoC4H3*, *Co4CL1*, *Co4CL2*, *CoF3′H1*, *CoLAR1*, *CoLAR2*, *CoLAR3*, *CoANR2*, and *CoANRL2* were significantly upregulated, while after 500 μM ABA treatment the expressions of *CoPAL3*, *CoCHS1*, *CoCHS4*, *CoF3′H1*, *CoDFR*, *CoLAR1*, *CoLAR2*, *CoLAR3*, *CoANS1*, *CoANR1*, and *CoANR2* increased dramatically. These results indicate that appropriate concentrations of ABA and MeJA activate C and EC biosynthesis and promote their accumulation in fruit shells. Our results provide new ideas and guidance for promoting the resource utilization of *C. oleifera* fruit shells.

## 1. Introduction

*Camellia oleifera* Abel, a shrub or small tree, belongs to the genus *Camellia* (Theaceae), and is one of the four major woody oil plants in the world [1]. As one of the most valuable woody oily plants, *C. oleifera* has a cultivation history of over 2300 years in China [2]. The edible oil produced from *C. oleifera* is known as “Eastern olive oil” and is highly praised for its high content of unsaturated fatty acids, which are instrumental in lowering blood pressure and cholesterol, protecting the liver, fighting cancer, and alleviating gastrointestinal discomfort [3,4]. Recently, the genomes of three types of *C. oleifera* have been sequenced and assembled, greatly accelerating research on this important woody oil crop [5].

The *C. oleifera* fruit shell refers to the skin that wraps around the seeds in the fruit, accounting for about 60% of the weight of *C. oleifera* fruit [6]. The high-quality edible oil extracted from seeds is the main product of *C. oleifera*. However, there are a large number of by-products after extracting edible oil, and the fruit shell is one of them [7]. These remaining fruit shells are mostly discarded or not fully utilized, which may be a burden on the environment [7]. So far, there have been research reports on using fruit shells to produce activated carbon, potassium carbonate, and deodorizers, or for extracting industrial chemicals such as tannins, xylitol, and furfural [7,8,9]. In addition, *C. oleifera* fruit shells also contain rich bioactive substances, which have good anti-inflammatory, bactericidal, and antioxidant effects and can be used in the field of healthcare [6,10].

Catechins are a class of phenolically active substances extracted from plants [11]. Catechin compounds mainly include the following eight monomers: catechin (C), epicatechin (EC), gallocachin (GC), epigallocatechin (EGC), catechin gallate (CG), epicatechin gallate (ECG), gallocachin gallate (GCG), epigallocatechin gallate (GCG), and epicatechin gallate (EGCG) [11,12]. The biological activities of catechins include anti-tumor, antibacterial, anti-inflammatory, antioxidant, antiviral, etc. [11,13]. Catechins belong to the flavonoid class and are produced through the phenylpropanoid metabolic pathway [13]. However, research on the biosynthesis and regulation of catechins mainly focuses on *Camellia sinensis*, and there are few reports in *C. oleifera*.

Exogenous application of methyl jasmonate (MeJA) can promote the production of flavonoid metabolites by affecting the upregulation or downregulation of gene expression in the flavonoid metabolism pathway [14,15]. After treating *Camellia vietnamensis* with MeJA for 2 h, the key genes involved in flavonoid metabolism pathways, such as *PAL*, *4CL*, *CHI*, *CHS*, *C4H*, *FLS*, etc., are found to have high expression levels [16]. Abscisic acid (ABA) significantly promotes the transcription of *NtFLS*, *NtF3H*, *NtF3H* and *NtANR* genes, as well as the accumulation of naringin, quercetin, isorhamnosum, kaempferol, and catechins in the flavonoid metabolite biosynthesis pathway [17]. In addition, there have been reports on the effects of other exogenous hormones on flavonoid accumulation. For example, gibberellin A3 (GA_3_) has been proven to be involved in regulating flavonoid metabolism during grape development [18], while salicylic acid (SA) can promote the total flavonoid content and biomass accumulation in *Phellinus igniarius* [19].

With the widespread promotion and cultivation of *C. oleifera* in China, the resource utilization of *C. oleifera* fruit shells has also received a lot of attention. However, due to the lack of effective resource utilization methods, a large number of *C. oleifera* fruit shells are still abandoned or directly burned, and the *C. oleifera* industry is still waiting for more efficient and environmentally friendly solutions. Improving the resource utilization of a large amount of by-product fruit shells will bring higher economic benefits to the *C. oleifera* industry. Interestingly, we found that fruit shells contain catechins, and spraying with a certain concentration of ABA and MeJA increased the accumulation of catechins in fruit shells, but the mechanism was still unclear. Therefore, this study aims to explore whether and how exogenous ABA and MeJA affect the accumulation and metabolism of catechins in fruit shells, providing a theoretical basis for ABA and MeJA to regulate the accumulation and metabolism of catechins in fruit shells of *C. oleifera*.

## 2. Results

### 2.1. The Fruit Shells of C. oleifera Contain Various Catechins

**Figure 1:**
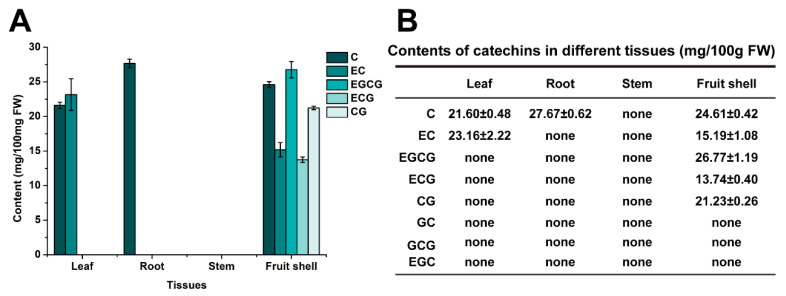
The types and contents of catechins in different tissues of *Camellia oleifera*. (**A**) Detection of different catechins in leaf, roots, stems, and fruit shells of *C. oleifera.* C: catechin, EC: epicatechin, GC: gallocachin, EGC: epigallocatechin, CG: catechin gallate, ECG: epicatechin gallate, GCG: gallocachin gallate, GCG: epigallocatechin gallate, EGCG: epicatechin gallate. (**B**) Statistical table for the detection of eight types of catechins in different tissues of *C. oleifera*. The error bars represent the standard deviation of three biological replicates.

During the ripening stage of *C. oleifera* ‘Huashuo’ (HS) fruit, catechin compounds were detected in the shells, roots, stems, and leaves. The results showed that the tissue of *C. oleifera* contained eight types of catechin compounds, including C, CG, EC, ECG, GC, GCG, EGC, and EGCG. However, three types of catechin compounds, including GC, GCG, and EGC were not detected (Figure 1A,B). The HPLC results showed that no catechins were detected in the stem of *C. oleifera*. The root only contained C, with a content of approximately 27.67 mg/100 g. The leaf contained 21.60 mg/100 g C and 23.16 mg/100 g EC. The contents of C, EC, CG, ECG, and EGCG in the fruit shells were detected, reaching 24.61, 15.19, 26.77, 13.74, and 21.23 mg/100 g, respectively. These results suggested that the shells of *C. oleifera* contains various catechins, which have great potential for utilization in health care.

### 2.2. ABA and MeJA Significantly Increased the Content of C and EC in C. oleifera Fruit Shells

**Figure 2:**
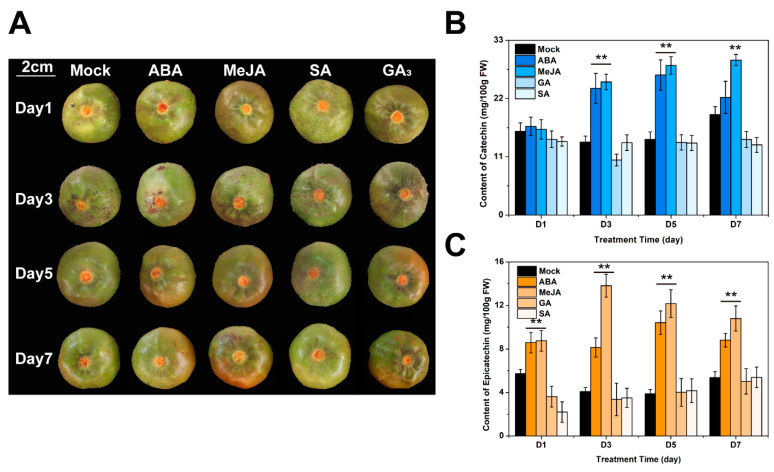
The effect of different hormones on the accumulation of C and EC in *C. oleifera* fruit shells. (**A**) The epigenetic changes of fruits after Abscisic acid (ABA), methyl jasmonate (MeJA), gibberellin A3 (GA_3_), and salicylic acid (SA) treatment. (**B**) Detection of C content under ABA, MeJA, GA_3_, and SA treatment. (**C**) Detection of EC content under ABA, MeJA, GA_3_, and SA treatment. The error bars represent the standard deviation of three biological replicates (**: *p* < 0.01).

To explore the accumulation pattern of catechin compounds in *C. oleifera* fruit shells under exogenous hormone treatment and provide a basis for production application, four different hormones (ABA, MeJA, GA_3_ and SA) were used to treat *C. oleifera* fruits for 7 days (Figure 2A). Fruit shells of *C. oleifera* were used for the extraction and detection of C and EC on the 1st (D1), 3rd (D3), 5th (D5), and 7th (D7) day. The results showed that ABA and MeJA significantly induced the accumulation of C in fruit shells. When treating with ABA, the accumulation of C in the fruit shells significantly increased on D3 (23.92 mg/100 mg) and D5 (25.14 mg/100 mg). The MeJA treatment group also showed a significant effect on promoting the accumulation of catechins, with a significant increase in the content of C in the fruit shells on D3, D5, and D7 (Figure 2B). The content of C in the fruit shells reached 25.14, 28.24, and 29.25 mg/100 g on D3, D5, and D7. However, after treatment with ABA and MeJA, there was no significant increase in C. In addition, compared with the mock group, the treatment with GA_3_ and SA did not result in significant changes in the content of C. Our results also showed that ABA and MeJA significantly increased the accumulation of EC (Figure 2C). After ABA treatment, the content of C in the fruit shell reached 8.58, 8.14, 10.42, 8.81 mg/100 g on D1, D3, D5 and D7, respectively. The content of C in the MeJA treatment group increased to 8.75, 13.81, 12.16, and 10.80 mg/100 g. On D1 of the ABA or MeJA treatment, the content of EC in the fruit shells significantly increased, indicating that the accumulation of EC was more sensitive to the influence of ABA and MeJA than C. Compared to ABA, MeJA was better at promoting the accumulation of EC.

### 2.3. The Biosynthesis of C and EC in C. oleifera Fruit Shells Was Significantly Promoted with 500 μM ABA

**Figure 3:**
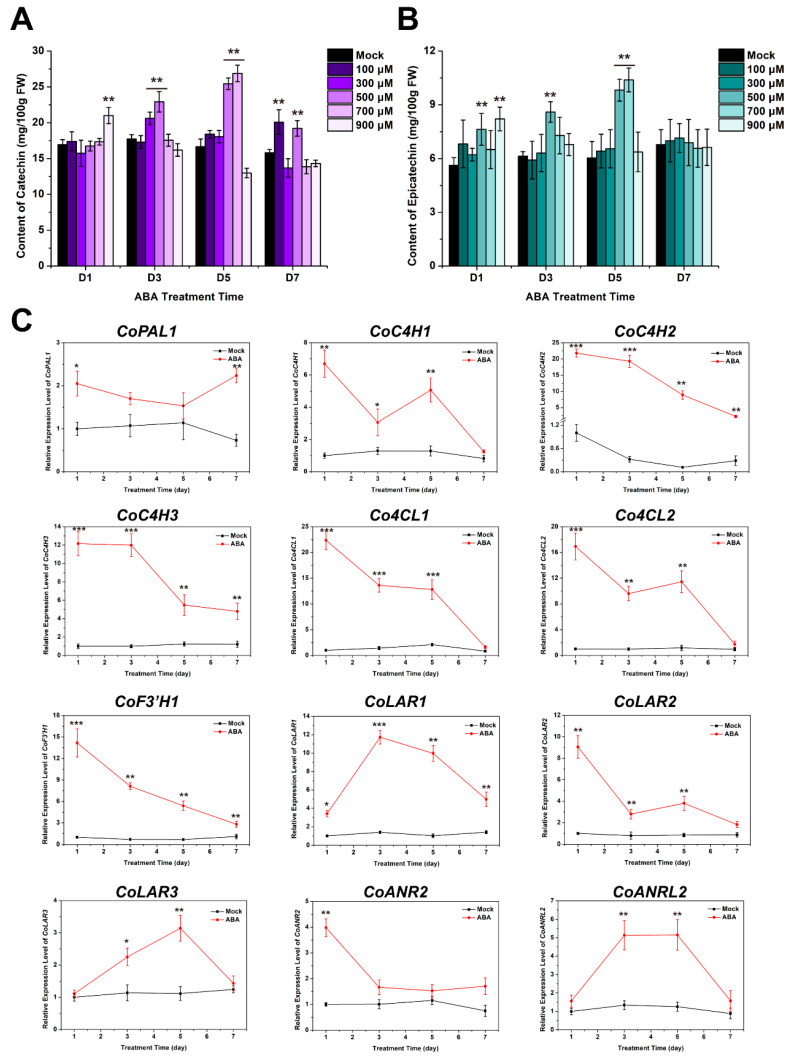
Effects of ABA on C and EC accumulation in *C. oleifera* shells. (**A**,**B**) Detection results of C and EC in fruit shells under different concentrations of ABA treatment. (**C**) Expression detection results of genes involved in catechin and epicatechin biosynthesis induced by 500 μM ABA. The error bars represent the standard deviation of three replicates (*: *p* < 0.05; **: *p* < 0.01; ***: *p* < 0.01).

In order to further investigate the optimal concentration of ABA in enhancing the accumulation of catechins in *C. oleifera* fruit shells, different ABA solutions of 100, 300, 500, 700 and 900 μM were used to treat *C. oleifera* fruits, and then the concentration of C and EC in the fruit shells was detected. The results showed that the 500 μM ABA solution is the most effective in enhancing the accumulation of C and EC (Figure 3A). Compared with D1, the concentration of C in the fruit shells on D3 (22.38 mg/100 g) was significantly higher than that of the control group (17.74 mg/100 g), which increased by about 20%. The concentration of C (25.52 mg/100 g) increased by about 53% compared to the control on D5. After treatment with a 300 μM ABA solution, the accumulation of C in the fruit shells significantly increased only on D3. There was a significant increase in C in the fruit shells on D7 after treatment with 700 μM ABA. In addition, the content of EC increased evidently compared to the control under 500 and 700 μM ABA treatment, with an increase of about 63% and 72%, respectively. However, from D3 to D7 following 900 μM ABA treatment, the levels of C and EC significantly decreased. Furthermore, the expression levels of genes involved in the biosynthesis pathways of C and EC were detected after 500 μM ABA treatment. The results of qRT-PCR showed that the expression levels of the flavonoid biosynthesis pathway genes, including *CoPAL1*, *CoC4H1*, *CoC4H2*, *CoC4H3*, *Co4CL1*, *Co4CL2*, and *CoF3′H1*, were obviously increased in fruit shells. *CoLAR1*, *CoLAR2* and *CoLAR3*, the enzymes to catalyze C biosynthesis, were significantly enhanced. Meanwhile, we also found *CoANR2* and *CoANRL2* showed higher expression levels after induction with 500 μM ABA. These results suggested that ABA might induce these genes’ expression to promote the accumulation of C and EC.

### 2.4. MeJA Effectively Increased the Transcription Level of Related Genes to Promote the Accumulation of C and EC in C. oleifera Fruit Shells

**Figure 4:**
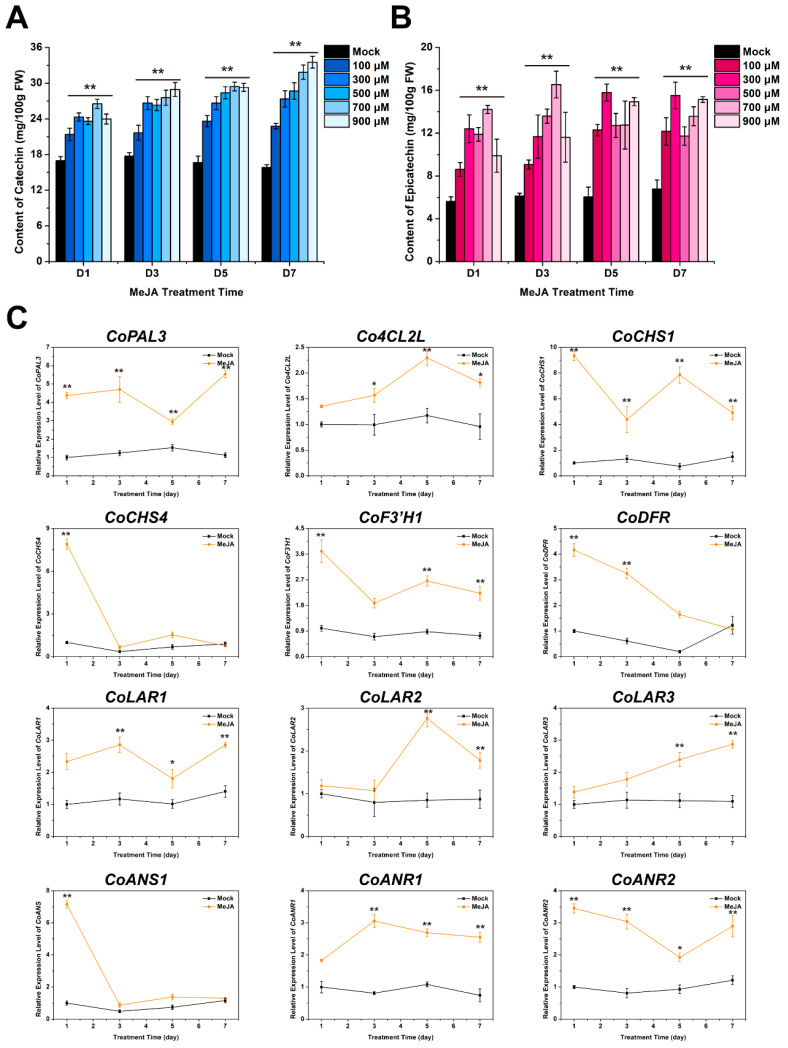
Effects of MeJA on catechins accumulation in *C. oleifera* shell. (**A**,**B**) Dynamic changes in the content of catechin and epicatechin in fruit shells treated with different concentrations of MeJA. (**C**) Expression detection results of genes involved in catechin and epicatechin biosynthesis induced by 900 μM MeJA. The error bars represent the standard deviation of three replicates (*: *p* < 0.05; **: *p* < 0.01).

In order to further investigate the optimal concentration of MeJA, we treated *C. oleifera* fruits with five different concentrations of MeJA. The results showed that 100, 300, 500, 700, and 900 μM MeJA could increase the content of C in fruit shells (Figure 4A). When 100 μM MeJA was used to treat fruit shells for 5 days, the content of C reached a peak of 23.61 mg/100 g. After treatment with the other four concentrations of MeJA, the peak concentrations of C were detected on D7 (29.15, 28.68, 31.85, 33.52 mg/100 g). In addition, the detection results of *C. oleifera* fruits for the 900 μM MeJA treatment on D7 showed that the C in the fruit shells reached 33.52 mg/100 g, an increase of about 98% compared to the control. These results indicated that the accumulation of C in the fruit shells gradually increased with the increase of MeJA treatment concentration over time. After 7 days of 900 μM treatment, the increase in C content in the fruit shells was the highest. Meanwhile, the content of EC in the fruit shells after MeJA treatment was also detected (Figure 4B). With the treatment of 100 and 300 μM MeJA, the concentration of EC increased with the peak concentration on D5 (12.29 and 15.79 mg/100 g), increasing by 118% and 180%, respectively. After 3 days of treatment with 500 and 700 μM MeJA, the EC concentration reached its peak (13.59 and 16.54 mg/100 g), with an increase of 141% and 193%, respectively. The content of EC reached 15.14 mg/100 g, an increase of 169% compared to the control group, after treatment with 900 μM MeJA for 7 days. Our results showed that the accumulation of EC was significantly promoted by MeJA, especially 300 μM MeJA. To further investigate the mechanism of MeJA promoting the accumulation of C and EC in fruit shells, we tested the expression levels of key enzyme genes involved in C and EC biosynthesis pathway under the 900 μM MeJA treatment. The results showed that *CoPAL3*, *Co4CL2L*, *CoCHS1*, *CoCHS4*, *CoF3′H1*, *CoDFR*, *CoLAR1*, *CoLAR2*, *CoLAR3*, *CoANS*, *CoANR1*, and *CoANR2* were induced to varying degrees by 900 μM MeJA (Figure 4C). Taken together, these results indicated that MeJA promoted the accumulation of C and EC in fruit shells by inducing the expression of key enzyme genes.

## 3. Discussion

*C. oleifera* is a woody plant with economic value in China, and the main compounds isolated from its seeds can be used as edible oil, which contains abundant unsaturated fatty acids [20]. The *C. oleifera* has a longstanding history of over 2300 years in China as a source of edible oil, with the focus on the content and quality of its seed oil consistently ranking as the top priority in breeding programs [21]. To meet the escalating demand for edible oil, the cultivation area for *C. oleifera* is steadily expanding. The production of *C. oleifera* fruit shells, a by-product of extracting edible oil, is also on the rise, potentially exerting significant environmental pressure. Consequently, there is an urgent need to develop resource utilization methods for these fruit shells.

### 3.1. C. oleifera Fruit Shells Can Be Used as a Potential Raw Material for Extracting Catechins

*C. oleifera* fruit shells, as a high-quality by-product, have gradually been developed and applied in many industries. The fruit shells contain tannins, furfural, bioactive phenolic compounds, and saponins, which are used to prepare chemical raw materials such as tannins, furan, and activated carbon [22,23,24]. In previous research, the fruit shells have been used to generate vanillin and ethanol, as well as xylooligosaccharides [25,26]. The extract of *C. oleifera* fruit shells is also used as a skin whitening agent in cosmetics [27]. *C. oleifera* fruit shells seem to be toxic to fungi and termites, indicating their potential as green pesticides [28].

Catechins are a class of natural polyphenolic compounds that are beneficial to human health, belonging to the flavan-3-ols (a subclass of flavonoids) [29]. Catechin is widely present in various fruits, vegetables, and plant beverages. For example, fresh tea, broad beans, rock-rose leaves, strawberries, and red wine all contain high concentrations of catechins [29]. However, there are still few studies on *C. oleifera*, especially on the fruit shells. In this study, eight types of catechins were detected in four types of *C. oleifera* tissues (roots, stems, leaves, and fruit shells). No catechins were found in the stem, while only C was found in root (Figure 1A,B). Compared to other organizations, we found that fruit shells contained more catechins compound such as C, EC, EGCG, ECG, and CG (Figure 1A,B). These results indicate that fruit shells of *C. oleifera* have greater potential value as raw material for extracting catechins.

### 3.2. ABA and MeJA Seem to Have a More Significant Effect on the Improvement of Catechins in C. oleifera Fruit Shells than Other Plant Hormones

The biosynthesis of flavonoids in plants is regulated by various factors, including abiotic factors such as temperature, light, and nutrition, as well as biotic factors such as pathogens and pests [1,30]. Phytohormones including auxin, gibberellin (GA_3_), abscisic acid (ABA), ethylene, and methyl jasmonate (MeJA) are one of the most critical regulatory factors for flavonoid biosynthesis in plants [1,18,19,31,32]. In this study, ABA, MeJA, GA_3_, and SA were used to treat *C. oleifera* fruits in order to increase the production of C and EC in fruit shells. We observed some color changes on the fruit shells treated with ABA and MeJA (Figure 2A). The content of C and EC in the fruit shell was measured on D1, D3, D5, and D7 after treatment. Interestingly, our results showed a significant increase in the content of C and EC after treatment with ABA and MeJA, which persisted until D7 (Figure 2B,C).

Our results found that the content of C in fruit shells showed significant improvement on D3 and D5 under ABA induction. The content of C increased by 73% and 85%, respectively, compared to the control (Figure 2B). More clearly, after MeJA treatment, the accumulation of C in the fruit shells increased by 82%, 98%, and 54% on D3, D5, and D7, respectively (Figure 2B). Figure 2C showed that on D1, D3, D5 and D7, the content of EC in the fruit shells of the ABA and MeJA treatment groups was significantly higher than that of the control group and the GA_3_ and SA treatment groups. Compared to the control group, the ABA treatment group showed an increase of 50%, 99%, 169%, and 64% in the EC content of the fruit shells on D1, D3, D5, and D7, respectively. Similarly, the MeJA treatment group exhibited increases of 52%, 238%, 214%, and 101% in the EC content compared to the control group in the same time periods. These results confirm that both ABA and MeJA effectively increased the content of C and EC in fruit shells, with MeJA having a more significant effect on increasing the accumulation of C and EC in fruit shells.

In addition, the accumulation of C showed a significant increase on D3 after ABA and MeJA treatment, while the content of EC significantly increased on D1. These results indicate that the increase in EC seems to be more sensitive to the effects of ABA and MeJA than the promotion of C. In addition, whether the color changes of fruit shells are related to changes in the content of C and EC remains to be further analyzed in the future.

### 3.3. The 500 μM ABA and 900 μM MeJA Treatments May Be the Most Suitable Phytohormone Concentrations in Current Research for Enhancing the Accumulation of C and EC in Fruit Shells

ABA and MeJA, as two important plant hormones, were shown to promote the biosynthesis and accumulation of C and EC in fruit shells by our data. We further investigated the effects of different concentrations of ABA and MeJA on the content of C and EC in fruit shells. In our results, the condition of 500 μM ABA treatment for 5 days is the best for improving the C and EC content in fruit shells in the ABA treatment group (Figure 3A,B). However, excessive treatment time or concentration of ABA is not conducive to the accumulation of C and EC. In the MeJA treatment group, 900 μM MeJA treatment for 7 days was the best for improving the C and EC content in fruit shells. This indicates that different hormones have different promoting effects on the biosynthesis of C and EC in fruit shells. Therefore, we believe that 900 μM MeJA seems more suitable as a strategy for enhancing C and E in fruit shells. Furthermore, we investigated the mechanisms by which 500 μM ABA and 900 μM MeJA promote the biosynthesis and accumulation of C and EC. PAL and C4H are important enzymes that allocate a large amount of carbon from phenylalanine to the biosynthesis of several important secondary metabolites. The biosynthesis of C is positively correlated with the expressions of *CsPAL* and *CsC4H* [33]. PAL, 4CL, and ANS have been shown to participate in flavonoid secondary metabolism in *Salvia miltiorrhiza* [34,35]. F3′H plays an important role in the synthesis of flavonoids, polyphenols, and anthocyanins [36]. LAR and ANR play important roles in the biosynthesis pathway of proanthocyanidins [37]. In particular, the expressions of white *LAR* and *ANR* genes is highly positively correlated with most metabolites in the tannin biosynthesis pathway [38]. Our findings found that ABA significantly enhanced the expressions of *CoPAL1*, *CoC4H1*, *CoC4H2*, *CoC4H3*, *Co4CL1*, *Co4CL2*, *CoF3′H1*, *CoLAR1*, *CoLAR2*, *CoLAR3*, *CoANR2*, and *CoANRL2* (Figure 3C), which was consistent with previous results. These results confirm that ABA promotes the biosynthesis and accumulation of C and EC by inducing the expression of these key enzyme genes.

Previous studies indicate that MeJA can significantly enhance the expressions of *CHS*, *DFR, ANS*, *ANR*, and *LAR* to promote the accumulation of anthocyanins [1,39]. After 6 weeks of treatment, seeds treated with MeJA showed significantly increased expression levels of genes related to flavonoid biosynthesis, such as *DFR*, *FLS*, *F3H*, and *F3′H2*, compared to untreated seeds [1]. Consistently, we found that MeJA increased the expressions of *CoPAL3*, *CoCHS1*, *CoCHS4*, *CoF3′H1*, *CoDFR*, *CoLAR1*, *CoLAR2*, *CoLAR3*, *CoANS1*, *CoANR1*, and *CoANR2* (Figure 4C), which was not completely consistent with the results of ABA treatment group. LAR and ANR are key enzymes for the synthesis of C and EC, respectively [1,39]. The expressions of *CoLAR1*, *CoLAR2*, *CoLAR3*, and *CoANR2* were simultaneously induced by ABA and MeJA in our data, while the expressions of *CoANR1* and *CoANRL2* were only induced by MeJA and ABA, respectively. These results suggest that members of the CoANRs may regulate EC synthesis by participating in different signaling pathways. Compared with the slow increase under MeJA induction, the expression levels of *CoLAR1* and *CoLAR2* increased rapidly under ABA induction. The expression of *CoLAR3* increased slowly under ABA and MeJA treatment. The expressions of *CoF3′H1* and *CoANR2* were upregulated by both ABA and MeJA induction. The difference was that the expressions of *CoF3′H1* and *CoANR2* increased rapidly and then decreased and tended to be stable after ABA induction, while the expressions of *CoF3′H1* and *CoANR2* increased rapidly and remained stable after MeJA induction. These results indicate that ABA and MeJA can jointly induce varying degrees of expression of some C and EC biosynthesis related genes and can also individually induce the expression of some C and EC biosynthesis related genes, thereby promoting the biosynthesis and accumulation of C and EC in fruit shells. Our results provide a new perspective on using ABA and MeJA treatment as a strategy to improve the accumulation of catechins in fruit shells and lay the foundation for a deeper understanding of the molecular mechanism by which ABA and MeJA regulate catechins in fruit shells.

## 4. Materials and Methods

### 4.1. Plant Material

The experimental material was 7-year-old *C. oleifera* ‘Huashuo’ (HS) collected from the Haoyunwei *Camellia oleifera* Planting Professional Cooperative in Liuyang City, Hunan Province. In the preliminary experiment of exogenous hormone selection, three plants with similar tree shapes, strong growth, and a large number of fruits were selected for experimental treatment. After determining the types of exogenous hormones, six other HS plants with similar tree shapes, healthy growth, and a large number of fruits were selected for experimental treatment. The roots, stems, leaves, and fruit shells of HS were collected in October, during the fruit ripening period, and frozen in liquid nitrogen immediately for temporary storage. The samples were ground into particles using liquid nitrogen and stored at −80 °C in an ultra-low temperature freezer for later testing.

### 4.2. Exogenous Hormone Treatment

The concentrations of ABA, MeJA, SA were 500 μM, while the concentration of GA_3_ was 300 mg/L. To increase the solubility of the hormones, 50 mL of ethanol was added when preparing the hormone solutions. For the control group, a 5% ethanol aqueous solution was used. There were 5 treatments, including the above 4 hormones and the control group, with 4 fruits in each hormone treatment group. Cloth strips were dipped in different hormone solutions and were used to apply the different hormones to the fruit shells. Fresh bags were used to wrap the fruits treated with hormones. On the 1st (D1), 3rd (D3), 5th (D5), and 7th (D7) day after hormone application, the fruits from various treatments were collected, peeled off their shells and stored in liquid nitrogen. The samples were then brought back to the laboratory and were ground into particles using liquid nitrogen. Then samples were stored in a −80 °C freezer until extraction and detection of catechins.

In addition, we configured 100, 300, 500, 700, and 900 μM ABA and MeJA solutions to further confirm the optimal hormone concentration that can enhance catechins in fruit shells. The method of collection and detection of samples was as described in the above paragraph.

### 4.3. Comparative Analysis of Catechin Types in Different Tissues of C. oleifera Samples

For comparative analysis of catechin types, the roots, stems, leaves, and shells of HS were collected in October, during the fruit ripening period, and numbered. After collection, they were immediately put into liquid nitrogen, brought back to the laboratory, ground into a granular form in a liquid nitrogen environment, and then stored at −80 °C in an ultra-low temperature freezer until HPLC testing.

### 4.4. Extraction of Catechins

The fresh HS fruit shells were ground and crushed under low-temperature liquid nitrogen freezing and stored in a −80 °C ultra-low temperature freezer to prevent oxidation. For analysis, 1 g (accurate to 0.0001 g) of the sample was accurately weighed and placed in a 50 mL centrifuge tube. Immediately, 10 mL of 95% ethanol solution was added and the sample was mixed evenly. The centrifuge tube was sealed and left to stand for 12 h for extraction. Every 12 h, the extraction solution was poured out into a new 50 mL centrifuge tube, and 10 mL of 95% ethanol solution was added to the residue for further extraction. The extraction was repeated 4 times, and the extraction solution was combined and 95% ethanol was added to reach a total volume of 50 mL. After centrifugation at 4000 rpm/min for 5 min, the supernatant was taken and a 0.45 μm organic phase microporous filter membrane was used for filtration.

### 4.5. Detection of Catechins in C. oleifera Fruit Shells by High Performance Liquid Chromatography (HPLC)

The mobile phase was 0.1% acetic acid water and acetonitrile. The extract of catechins was separated by gradient elution detected by using Athena C18-WP (4.6 × 250 mm, 5 μm) chromatographic column (Shanghai Anpu Experimental Technology Co., Ltd., Shanghai, China) with a column temperature of 30 °C, 1.0 mL/min flow rate, and 10 μL injection. The detection wavelength was 278 nm. Flavonoid compounds were detected by gradient elution using mobile phase A, consisting of 100% acetonitrile, and phase B, consisting of water containing 0.1% acetic acid, at a 1.0 mL/min flow rate and a column temperature of 35 °C.

### 4.6. Gene Expression Analysis by Quantitative Real-Time Polymerase Chain Reaction (qRT-PCR)

The qRT-PCR experimental steps were performed according to the ChamQ Universal SYBR qPCR Master Mix special premix (Q711) reagent manual of Vazyme: pre denaturation at 95 °C for 30 s, 95 °C for 10 s, 60 °C for 30 s, 40 cycles. Experimental data analysis was conducted using the 2^−ΔΔCT^ method.

### 4.7. Data Statistics and Analysis

The data statistics and analysis were conducted using Excel 2019 software, and the results were presented as mean ± standard deviation. Student’s *t*-test was used to detect significance, and the STDEV formula was used to calculate the error. The data were graphed using Origin 8.0.

## 5. Conclusions

This study found that *C. oleifera* fruit shells have potential value as a raw material for the extraction of catechins. We analyzed the changes in the content of C and EC in fruit shells treated with four different plant hormones and focused on exploring the optimal concentrations of ABA and MeJA. Our results showed that 500 μM ABA and 900 μM MeJA had a significant effect on promoting C and EC in *C. oleifera* fruit shells. Furthermore, we confirmed that ABA and MeJA promoted the biosynthesis and accumulation of C and EC by inducing the expression of key enzyme genes involved in their biosynthesis. Among them, *CoLAR1*, *CoLAR2*, *CoLAR3*, *CoF3′H1*, and *CoANR2* were simultaneously induced by both ABA and MeJA, indicating that these genes played a more important role in phytohormone induced C and EC biosynthesis. Our results provide new ideas and strategies for the resource utilization of *C. oleifera* fruit shells.

## Data Availability

All data generated or analyzed during this study are included in this article.
